# Laparoscopic cholecystectomy in acute gallstone pancreatitis in index hospital admission: feasibility and safety

**DOI:** 10.12669/pjms.303.4380

**Published:** 2014

**Authors:** Ahmed Khan Sangrasi, BM Syed, Amir Iqbal Memon, Abdul Aziz Laghari, K. Altaf Hussain Talpur, Jawaid Naeem Qureshi

**Affiliations:** 1 **Ahmed Khan Sangrasi, FCPS, Liaquat University of Medical & Health Sciences, Jamshoro, Sindh, Pakistan.**; 2 **BM Syed, PhD, Liaquat University of Medical & Health Sciences, Jamshoro, Sindh, Pakistan.**; 3 **Amir Iqbal Memon, FCPS, Liaquat University of Medical & Health Sciences, Jamshoro, Sindh, Pakistan.**; 4 **Abdul Aziz Laghari, FRCS, Liaquat University of Medical & Health Sciences, Jamshoro, Sindh, Pakistan.**; 5 **K. Altaf Hussain Talpur, FCPS, Liaquat University of Medical & Health Sciences, Jamshoro, Sindh, Pakistan.**; 6 **Jawaid Naeem Qureshi, FRCS, Liaquat University of Medical & Health Sciences, Jamshoro, Sindh, Pakistan.**

**Keywords:** Cholecystectomy, Laparoscopy, Pancreatitis

## Abstract

**Background and Objective:** Acute gallstone pancreatitis is quite common throughout the globe. Conventionally definitive cholecystectomy has been delayed in index hospital admission. Since the last decade timing of cholecystectomy is gradually shifting towards the earlier phase of disease and currently gallstone pancreatitis is being evaluated as a further indication for laparoscopic cholecystectomy. There is also great concern regarding compliance of patients for definitive surgery due to poverty, ignorance and illiteracy in developing countries. The aim of this study was to assess the feasibility and safety of laparoscopic cholecystectomy as a definitive treatment in patients with mild and resolving gall stone pancreatitis.

**Methods:** This was a prospective study from July 2009 to June 2012. Patients were diagnosed by clinical examination, biochemical tests, ultrasonography and contrast enhanced CT. Patients with mild form of the disease (Ranson Score ≤3) and who showed clinical improvement were offered laparoscopic cholecystectomy in index hospital admission. Those who were unfit for surgery were referred for endoscopic sphincterotomy. Common bile duct stones were excluded preoperatively.

**Results:** A total of 38 patients were admitted with acute gallstone pancreatitis in the study period. The mean age of patients was 46.3 years with male to female ratio of 11/27. 22 (57.8%) patients were selected for laparoscopic cholecystectomy and procedure was completed successfully. Ten (26.3%) patients were referred for ERCP and endoscopic sphincterotomy and 11 (28.9%) were managed by conservative treatment and went without any definitive treatment. Mean duration of time from onset of symptoms and laparoscopic cholecystectomy was 7 days (range 4-10). Mean duration of operative time was 45 minutes and hospital stay was 7 days. There was no operative mortality. No major intra-operative or post-operative complication was recorded. two patients (9%) had minor complications.

**Conclusion:** Laparoscopic cholecystectomy can be safely performed in selected cases of mild gallstone pancreatitis in order to prevent further attacks of acute pancreatitis and other consequences of delayed treatment. Furthermore it resolves the problem of noncompliance of patients in third world countries where many patients are lost for definitive treatment.

## INTRODUCTION

Acute pancreatitis secondary to gall stone still remains a challenge to clinicians despite achieving the highest standards of care in the present era. Gallstones account for 30-50% cases as an etiological factor.^1^^-^^3^ Till yet, no consensus has been built regarding the definitive treatment for acute pancreatitis, however many developments in the recent past have at least led us to a comparatively better understanding and management of this clinical scenario. Surgical removal of gall bladder and clearance of biliary tree or the essential steps in the management in order to prevent further attacks.

Laparoscopic cholecystectomy (LC) and intra-operative cholangiography is the most preferred modality of treatment currently.^4^^,^^5^ Appropriate timing of cholecystectomy in the patients with acute gallstone pancreatitis is still controversial.^6^^-^^8^ Beside this there is also a great concern regarding compliance of patients for definitive surgery due to poverty, ignorance and illiteracy specially in developing countries. The aim of this study was to assess the feasibility and safety of laparoscopic cholecystectomy as a definitive treatment in patients with mild and resolving gall stone pancreatitis.

## METHODS

This was a prospective study from July 2009 to June 2012 at department of surgery Liaquat Univeristy of Medical & Health Sciences, Jamshoro and a private hospital at Hyderabad Sindh, Pakistan. Diagnosis of gallstone pancreatitis was established according to our hospital protocol. On clinical examination patient having pain in epigastrium with cholelithiasis on ultrasound with or without stones in common bile duct (CBD). Findings of pancreatic edema on ultrasound or CT scan, raised pancreatic enzymes (serum amylase) above three times than normal, raised alanine aminotransferase (ALT) by three times or more. Alcohol induced acute pancreatitis was excluded by history. When patient revealed alcohol consumption of >40 grams daily and biliary stone disease was excluded by ultrasound, pancreatitis was assumed to be due to alcohol. Other causes of acute pancreatitis like drugs, hyperlipidemia, ERCP (endoscopic retrograde cholangiopancreatography), post-operative and idiopathic were labeled accordingly and excluded from study. Severity of the disease was graded for selection purpose according to ranson’s criteria.^9^ Mild acute biliary pancreatitis (ABP) was categorized by patients with ranson’s score <3, while patients with score >3 were considered as severe disease. These patients with severe disease were excluded from study and treated conservatively. 

Early laparoscopic cholecystectomy was considered for patients operated between time of attack to within 10 days. All patients were counseled in detail and written consent was obtained. Biliary tree clearance was achieved either prior to surgery by ERCP or per-operatively by choledochoscopy. All patients underwent laparoscopic cholecystectomy in standard way using four ports. Pneumoperitoneum was created by placing first 10mm port using open Hasson technique infraumblically. Another 10mm port was placed in epigastrium. Two 5mm ports were placed in mid line and right subcostal region. Additional ports were placed according to requirement. Dissection was performed by diathermy and harmonic scalpel was used in difficult dissections in calots area. Subhepatic drain was placed in patients in whom difficult dissection was encountered and either bleeding or bile leakage was anticipated. Operative and postoperative parameters like mean operative time, number of ports, mean hospital stay, placement of drain and post-operative complications were recorded and analyzed. All operations were performed by the same surgeon. The patients were followed after a week first time, then monthly and minimum follow-up period was three months.

## RESULTS

Mean age of patients was 46.3 years with male to female ratio of 11/27. Out of 38 patients admitted with acute gallstone pancreatitis 22 patients were graded as mild disease according to our hospital protocol and were selected for definitive treatment. Sixteen patients with ranson’s score >3 were considered as severe disease and excluded from study. Ten out of sixteen patients with severed disease were found to have stone in common bile duct (CBD) were referred for endoscopic retrograde cholangiopancreatography (ERCP). Laparoscopic cholecystectomy was successful in 22 (57.8%) patients in index hospital admission within 4-10 (mean 7) days after admission. CBD dialatation of >6mm was recorded in 6 patients. 10/38 patients were referred for ERCP. Five of these patients for management purpose as they were having severe disease and were excluded from our study. Remaining five patients were referred for clearance of biliary tree prior to laparoscopic surgery. Duration of operation varied between 25-55 (mean 45) minutes. In majority of patients 18/22 (81.8%) were operated using our standard four ports, 4/22 (22.7%) required an additional port making five ports in total. In 4 (18.1%) patients we encountered difficult dissection including fibrous adhesions of omentum with gall bladder and oedematous calots triangle. The average discharge time was 7 days ranging between 4-11 days. Drain was needed to be placed in 6 (27.2%) patients. There was no major ductal or vascular complication. Minor complication like port infection was observed in two (9%) cases. Operative mortality was zero.

## DISCUSSION

Considerable morbidity and mortality is associated with acute pancreatitis.^1^^,^^10^ After first attack, recurrence of biliary pancreatitis occurs in 25-76% of the cases.^11^^-^^14^ Many general surgeons treating the patients of acute biliary pancreatitis, probably would still choose to wait and perform an interval cholecystectomy after 6-8 week.^7^^,^^15^ The association between biliary stones and acute pancreatitis may be as old as development of gallstones, but first to report this fact were Bernard (1852) and Prince (1882).^16^ Than Opie^17^ in 1901 during autopsy of two patients who developed acute pancreatitis, reported impaction of gall stones in ampulla of vater.

**Table-I:** Demographics of Patients

No. of patients	38
Mean age (yrs.)	46.3
Male: Female ratio	11: 27
No. of patients receiving definitive treatment on index Hospital admission	22
No. of patients referred for ERCP for definitive treatment	5
No. of patients referred for ERCP prior to operation	5
No. of patients managed on conservative treatment or left without any definitive treatment	11

**Table-II:** Operative and Post-operative variables (n=22).

Mean duration of early laparoscopic cholecystectomy (days)	7 (range 4-10)
No. of ports: -4 -5	18 (81.8%) 4 (18.1%)
No. of patients with difficult dissection	4 (18.1%)
Mean operative time (minutes)	45 (Range 25-55)
Mean hospital stay (days)	7 (Range 4-11)
Placement of drain	6 (27.2%)
Complications - Major: - Vascular- Ductal- Minor	0 0 0 2 (9%)

Mortality rate in recurrent attacks is as high as 10% and rate has been observed upto 40%.^18^ Cholecystectomy has been proven to be the only definitive treatment, which reduces the risk of biliary pancreatitis to as low as zero.^12^^-^^14^^,^^18^ ERCP (Endoscopic retrograde cholangiopancreatography) and sphincterotomy are alternative modalities which decrease but does not completely eliminate the recurrence of pancreatitis. Furthermore, ERCP does not prevent other complications of cholelithiasis like acute cholecystitis.^19^^-^^23^

Migration of gallstones leading to obstruction of common bile duct (CBD) and pancreatic duct maybe a cause of activation for initiation of acute biliary pancreatitis.^24^ Despite the formal recommendations for early cholecystectomy in mild gallstone pancreatitis by many international hepato-biliary societies, many surgeons in the developing countries are still reluctant to perform early cholecystectomy. Probably this fear is due to their experience with complications of the gall stone pancreatitis and non-availability of the state of art facilities to manage this condition.

Early cholecystectomy either laparoscopic or open in the management of mild acute biliary pancreatitis is also advocated by other similar studies.^6^^,^^25^^,^^26^ On the other hand many studies^7^^,^^15^^,^^27^^-^^29^ have recommended deferring the surgery for interval cholecystectomy until 6-8 weeks, because they have recorded an increased procedure and anesthesia related morbidity and mortality. In this study we were successful to perform early cholecystectomy in all cases without any additional technical problem. The only disease related problem during laparoscopic cholecystectomy encountered by us was difficult retraction due to increased size of oedematous pancreas. This led to need of an additional 5^th^ port for retraction in order to push duodenum downwards.

The policy of cholecystectomy as a definitive treatment in the patients with acute biliary pancreatitis is driven from the fact of high rate of recurrence of disease if patients are left without definitive treatment.^7^ This issue is most relevant to third world and developing countries where ratio of defaulters for definitive treatment is too high. There are many reasons for that like illiteracy, poverty, ignorance and dearth of medical facilities. It has been observed in developing countries that inspite of detailed counseling and providing information regarding the gravity of consequences of the disease, follow up of the patients is very poor. This observation was also reflected in this study as none of the eleven patients who were managed conservatively returned for definitive cholecystectomy. Due to this poor follow-up for definitive treatment, we could not perform a comparative study between two groups. Therefore it is highly obligatory for surgeons of third world to make maximum efforts to provide definitive treatment of acute biliary pancreatitis in index hospital admission in order to decrease the number of such defaulters.

Previously it was considered that timing of cholecystectomy is not based only in the severity of pancreatitis but also on preference of the physician. Now several studies have suggested that surgeons should shift to early cholecystectomy in at least milder form of the disease. Our study also supports this viewpoint with insignificant complications during early cholecystectomy (Table-I). Now after a long experience of managing the disease we have a better understanding of the pathophysiology and natural course of the disease. Now surgeons believe that early surgery in acute biliary pancreatitis do not pose danger regarding any major complications related to anaesthesia and surgery. Nealon et al.^30^ has advocated deffering the surgery until 6-8 weeks in order to prevent the development of pseudocyst or necrosis of pancreas in postoperative period. Our study (Table-II) as well as other studies^31^ have shown laparoscopic cholecystectomy in milder form of acute biliary pancreatitis does not lead to development of such complications. There are some studies^32^^,^^33^ which suggest acute cholecystitis, thick walled gallbladder and nonfunctioning gallbladder resulting in technical difficulties during surgery. However we do not agree with these studies as these operative finding are the routine operative findings in laparoscopic cholecystectomies for gall stone disease. Therefore we do not consider these factors as a contributing factor to operative difficulty. We have concluded that in mild acute biliary pancreatitis one can safely manage the patient by early laparoscopic cholecystectomy. While patients with severe disease should be initially managed by conservative treatment, followed by interval laparoscopic cholecystectomy after 6-8 weeks. Moderate biliary pancreatitis still remains at border line of timing for definitive surgery. For this subset of patients, other factors like age, co-morbidities, preference of patient and judgment of attending physician will guide in relation to timing for appropriate definitive treatment. We also recommend appropriate fluid resuscitation and anti-biotic therapy for better outcome of this clinical scenario prior to laparoscopic cholecystectomy.

## CONCLUSION

Laparoscopic cholecystectomy can be safely performed in index hospital admission in mild gallstone pancreatitis in order to prevent further attacks of acute pancreatitis and other consequences of delayed treatment. Furthermore it solves problem of noncompliance of patients in third world countries where many patients are lost for definitive treatment.

## Author Contributions:

**AKS and BMS:** Conceived and designed the study, did statistical analysis.

**AIM and AAL:** Data collection and editing of manuscript.

**KAHT and JNQ:** Critical Review and final approval of the manuscript.
